# Sonographic examination at the beginning of the second stage of labor predicts birth outcome in vaginally intended breech deliveries: a blinded prospective study

**DOI:** 10.1007/s00404-023-07011-x

**Published:** 2023-03-24

**Authors:** Lukas Jennewein, Ricarda Heemann, Samira Catharina Hoock, Anna Elisabeth Hentrich, Christine Eichbaum, Susanne Feidicker, Frank Louwen

**Affiliations:** Department of Gynecology and Obstetrics, School of Medicine, Goethe-University, Theodor-Stern-Kai 7, 60590 Frankfurt, Germany

**Keywords:** Vaginal breech delivery, Ultrasound examination, Birth observation, Delivery outcome

## Abstract

**Purpose:**

In order to spread competence in vaginal breech deliveries, it is necessary to develop new and easily applicable tools for birth progression and safety evaluation. Ultrasound is a useful and ubiquitously available tool with already documented value for birth progression observation. In deliveries out of breech presentation, an established ultrasound examination is missing. We determined the descent of the fetal buttocks in relation to the maternal pelvic inlet using intrapartum ultrasound. We evaluated these results in comparison to the clinical vaginal examination with the aim to establish an easily applicable method for birth outcome prediction. Therefore, we analyzed the predictive value of our examinations on birth outcome parameters, such as cesarean section rate, as well as fetal and maternal outcome parameters.

**Methods:**

We performed a prospective blinded study on 106 mothers with vaginally intended breech delivery. At beginning of stage two in labor, the descent of the fetal buttocks into the mother’s pelvic inlet was detected with transabdominal ultrasound and vaginal examination by different observers. Primary outcome variable: Cesarean section rate. Secondary outcome variables: rate of manual assistance in vaginal deliveries, birth duration, 5′ APGAR score, umbilical arterial pH, maternal blood loss, and perineal injury. For non-parametric values, Wilcoxon’s *χ*^2^ test was performed. In order to analyze the predictive value of our examination, lack-of-fit analysis was conducted. Reliability evaluation of the sonographic examination was done with a matched-pair analysis.

**Results:**

Women with positive intrapartum ultrasound breech engagement sign (+ IPUBES) had a significantly lower rate of cesarean section in comparison with those with negative IPUBES (5/67; 7.5% vs. 18/39; 46.2%; *p* < 0.0001). The area under the ROC curve for the prediction of CS for negative IPUBES was 0.765 with a sensitivity of 78.3% and a specificity of 74.7%. Sonographic examination showed an excellent reliability in a matched-pair analysis comparing vaginal and sonographic examinations with a mean difference of 0.012 (SD ± 0.027, 95% CI − 0.014 to 0.065). Mean birth duration was significantly longer in deliveries with negative IPUBES (533 min vs. 440 min; *p* = 0.0011). Fetal and maternal outcome parameters were not significantly different between deliveries with positive and negative IPUBES.

**Conclusions:**

Sonographic evaluation of the fetal descent in relation to the mother’s pelvic inlet screens reliably for emergency cesarean section. This newly presented method for birth progression observation might be a powerful tool for distribution of expertise in vaginal breech delivery and is able to give reference for clinical vaginal examination by obstetricians in training.

**Trail registry:**

Clinical trial. Date of registration: 13.03.2019; Date of initial participant enrollment: 20.03.2019; DRKS00016885; https://www.drks.de; German clinical trials register.

## What does this study add to the clinical work

**Table:** 

A positive intrapartum ultrasound breech engagement sign (Fetus entered the maternal pelvic inlet) is associated with vaginal birth. Intrapartum ultrasound can be supportive and beneficial for obstetricians in training who are learning vaginal breech management.	

## Introduction

Fetal breech presentation in term pregnancies is one of the most common reasons for elective cesarean section. This is despite the fact that vaginal delivery is a safe option for most women. Numerous studies document the safety and benefit of vaginally intended deliveries [1–3]. High cesarean section rates worldwide lead to an increased maternal and fetal morbidity [4–8]. In consequence, various national guidelines recommend the vaginal birth approach in term pregnancies with breech presentation [9–11]. There is plenty discussion on the selection criteria upon which pregnant women are eligible for trial of labor when breech presentation occurs. The largest prospective cohort study collective on vaginally intended breech deliveries was able to show that parity, birth induction, fetal birth weight, birth after cesarean, and fetal leg posture impact emergency cesarean section rate but not fetal morbidity when deliveries are performed in an upright maternal position [12–17]. Still there are only few obstetrical centers offering vaginal birth out of breech presentation, depriving many women of the ability to choose their preferred intended birth mode. Management of vaginal breech delivery is easy to learn and can be implemented into clinical practice [3, 18] but presumably because of medico-legal reasons and low incidence of breech presentation leading to scarce expertise, there is barely any increase in numbers of obstetrical departments offering vaginal breech birth.

Additional diagnostical tools might lower barriers to implement new clinical procedures such as the vaginal breech delivery. Sonographic assessment is easily applicable and available almost in all obstetrical departments worldwide. In vertex deliveries, sonographic examinations already are established [19–27]. There are convincing data on the benefit and reliability of sono-graphical determination of fetal position [19, 20] and fetal descent [23]. The “occiput-spine angle” has prognostic value for predicting successful operative vaginal birth [22]. Also trans-perineal ultrasound is an established method for birth progression monitoring, measuring different parameters, such as the head direction, the progression distance, or the head perineum distance [23–27]. In deliveries out of breech presentation, evidence on intrapartum ultrasound is very limited. There is one study about the intra- and inter-observer variability of trans-perineal ultrasound evaluating the ‘breech progression angle’ on 44 patients [28]. In this study, the most inferior part of the fetus was detected. The most important fetal part on which birth progression monitoring should be assessed is the fetal pelvic bone/the fetal buttocks [17]. Studies analyzing ultrasound examination during birth in breech deliveries focusing on the fetal pelvis are not existing to our knowledge.

In order to address the need for intrapartum sonographic examination in breech delivery, we performed a single-center blinded prospective study using transabdominal ultrasound and compared vaginal examination and sonographic examination at beginning of the second stage of labor. Our goal was to establish a useful and easily applicable method during birth for delivery outcome prediction.

## Materials and methods

### Clinical procedure and sonographic examination

**Fig. 1:**
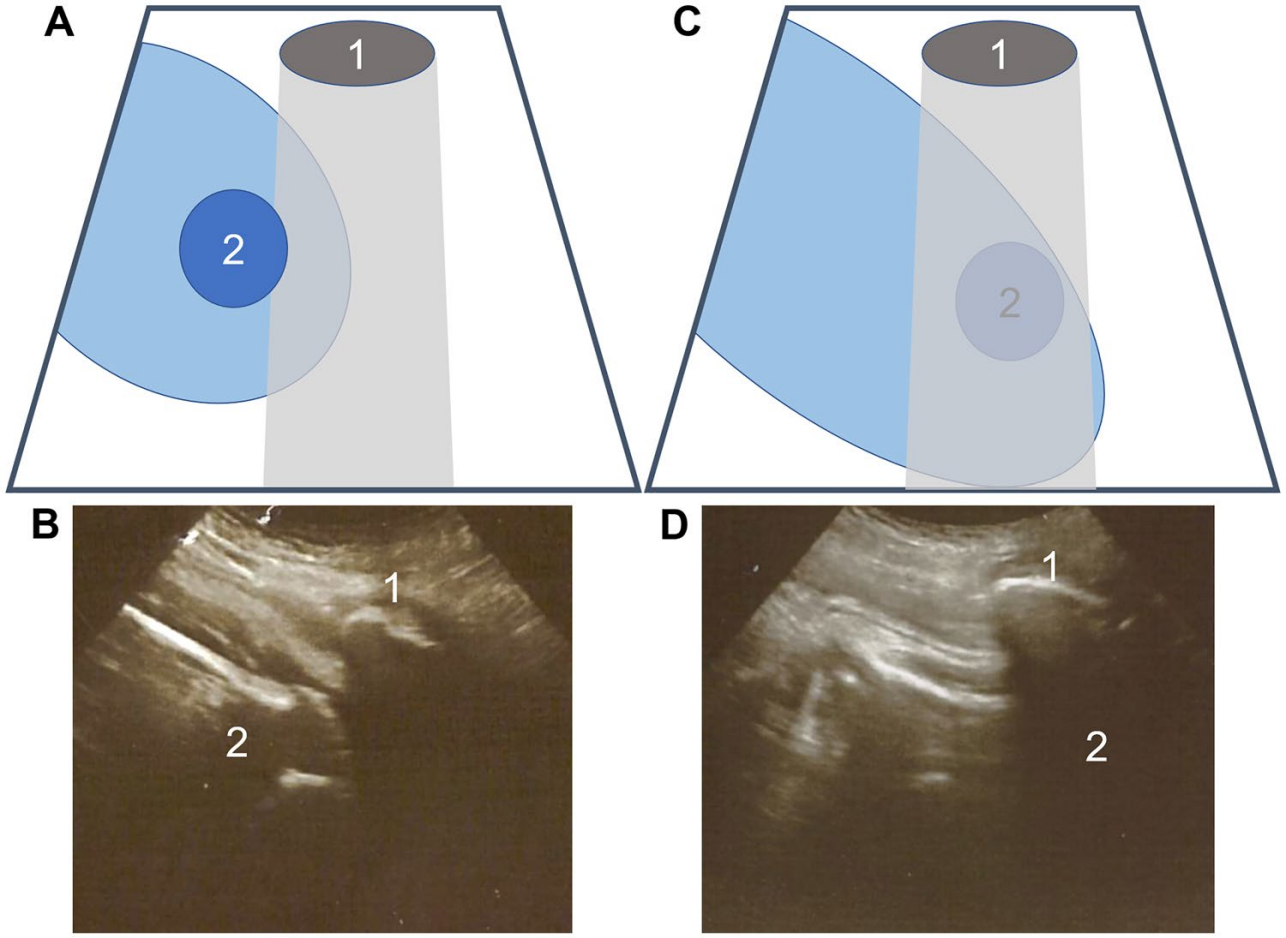
Sonographic examination results, **A** schematic and **B** sonographic example showing a negative intrapartum ultrasound breech engagement sign. The fetal pelvis is above the pelvic inlet of the mother. **C** Schematic and **D** sonographic example showing a positive intrapartum ultrasound breech engagement sign. The fetal pelvis has entered the maternal pelvic inlet. 1: Maternal symphysis or pubic crest, 2: fetal pelvic bone/hip

Patients who opted for vaginal delivery at term with a fetal breech presentation were recruited. Patients were counseled as a clinical standard protocol including the information about possible complications of a vaginal birth and a cesarean section. Primiparous women received an MRI in order to determine the obstetric conjugate as well as the intertuberous distance. Measurements were done by specialized radiologists. The counseling process includes the offering of an external cephalic version. All vaginal deliveries are performed on all fours or in upright position including manual assistance for arm (Louwen maneuver) or head delivery (Frank Nudge) if necessary as described in [15]. Vaginal breech delivery is supervised/managed by trained obstetricians who undergo a special training containing Skill’s-Lab-based training and the attendance as well as supervised management of breech deliveries [18]. At beginning of stage II (cervix fully dilated in the standard vaginal examination by the obstetrician), a second obstetrician performed an ultrasound examination on every participant in between contractions. The sonographic examination as well as the correct documentation was demonstrated to each observer prior to the study. Both results (sonographic and clinical pelvic exam) were documented separately and blinded. Both examinations take place in a supine position. The mother has to lie flat on her back. The transducer is placed perpendicular (90°) to the maternal abdomen, pointing straight downwards, visualizing the pubic crest or symphysis. If the fetal pelvic bone or trochanter major is visible above the symphysis, the fetal pelvis has not entered the maternal pelvic inlet. This is called the negative intrapartum ultrasound breech engagement sign (−IPUBES). If the pelvic bone or the upper fetal femur is visible below the maternal symphysis or fetal organs (e.g., kidney, bladder) are visible at the level of the maternal pelvic inlet, the fetal pelvis has entered the maternal pelvic inlet. This is the positive intrapartum ultrasound breech engagement sign (+ IPUBES) (see Fig. 1).

In clinical vaginal examination, the parameter of fetal pelvic descent (beneath or above the symphysis) was documented. The examination is performed in a maternal supine position as a standard also outside the study. Ultrasound examination was performed using a Voluson P8 ultrasound with a standard abdominal 4C convex transducer from GE Healthcare, Chicago, USA.

### Patient cohort and ethics committee approval

The actual Helsinki Declaration applied for this study. We performed a blinded prospective cohort intervention study on vaginally intended deliveries of term singletons in breech presentation (> 37 weeks of pregnancy) at the Goethe University Hospital in Frankfurt from 03/2019 to 12/2021. The university’s ethics committee gave consent (#355/18). The study was registered online in the German clinical trials register (#DRKS00016885). All patients gave written consent before birth. We recruited 129 patients. Exclusion criteria were planned elective cesarean section, estimated birth weight of below 2500 g, pregnancy duration of below 37 weeks, uterine malformation, diabetes, an intertuberous distance of below 11 cm, or lack of consent. Twenty-three patients were excluded because of missing/incomplete data and/or cesarean section in stage I of labor (9 cases). Indications were arrest in labor (3 cases), mother’s wish (2 cases), and non-reassuring fetal heart rate (4 cases).

### Data collection and preparation

Examination results of the sonographic and the clinical vaginal examinations were executed by different observers and documented separately enabling a blinded inquiry. There were 19 different observers. For all other data, the hospital’s patient management system was used for data acquisition. Data was merged and anonymized before analysis after all data were collected. Primary outcome parameter: cesarean section rate. Secondary outcome parameters: Manual assistance in vaginal delivery, birth duration, 5′ APGAR score, umbilical arterial pH, perineal injury, and maternal blood loss.

### Statistical analyses

Variables were tested if normal distribution applied (Kolmogorov–Smirnov testing). Pearson’s *χ*^2^-test was utilized to detect group differences. Specificity and Sensitivity were calculated with contingency tables of the respective examination. Post hoc analysis in our cohort revealed a Power of 97.2% (variable: negative intrapartum ultrasound breech engagement sign—outcome: cesarean section). Lack-of-fit analysis was performed in order to compare test accuracy between sonographic and vaginal examination. A matched-pair analysis comparing vaginal and sonographic examinations was conducted in order to evaluate reliability. Statistical analyses were done using JMP software (Version 14.0, SAS Institute, Cary, NC, USA). A *p *value of below 0.05 was considered as statistically significant.

## Results

One hundred and six patients were included in our analysis. Maternal and fetal descriptive parameters are displayed in Table 1. In 70 vaginal examinations (66.0%), the fetus’ buttocks entered the maternal pelvis at beginning of the second stage of labor (vaginal examination positive), whereas the result in sonographic examination was only in 67 cases (63.2%) positive (positive intrapartum ultrasound breech engagement sign, + IPUBES). Cesarean section rate in the study cohort was 21.7%, rate of manual assistance 31.1% (see Table 1).

**Table 1:** Study cohort

Variable	Cohort, *N* = 106
Age (mean ± SD)	32 ± 4.5
BMI (mean ± SD)	23.3 ± 4.4
Duration of pregnancy in weeks ± SD	39 ± 1
Parity (*n*, %)	
1	69 (65.1%)
2	30 (28.3%)
> 2	7 (6.6%)
Fetal birth weight (grams; mean ± SD)	3318 ± 407
Epidural anesthesia	67 (63.2%)
Vaginal examination positive	70 (66.0%)
Positive intrapartum ultrasound breech engagement sign (+ IPUBES)	67 (63.2%)
Delivery mode	
Spontaneous vaginal birth	50 (47.2%)
Manually assisted birth	33 (31.1%)
Cesarean section	23 (21.7%)

### Primary outcome: cesarean section rate

Cesarean section rate was significantly different between groups with a rate of 46.2% in −IPUBES and a rate of 7.5% in + IPUBES group with a *p* value of below 0.0001 (Table 2). We estimated the prognostic value of both vaginal and sono-graphical examinations to predict cesarean section when the examination was negative (fetal pelvis did not enter the maternal pelvic inlet). −IPUBES was associated with emergency cesarean section with a sensitivity of 78.3% in comparison to 69.6% sensitivity of negative vaginal examination. Specificity was 74.7% (sonographic examination) and 75.9% (vaginal examination). Negative predictive value was 92.5% (sonographic) and 90% (vaginal). In a lack-of-fit analysis, there was no significant difference in test accuracy when both tests were compared with a *p* value of 0.536 (negative vaginal examination result AUC 0.727; −IPUBES AUC 0.765) (see Table 3).

**Table 2:** Birth outcome related to sonographic examination result

Characteristic	Negative intrapartum ultrasound breech engagement sign (−IPUBES) *N* = 39	Positive intrapartum ultrasound breech engagement sign (+ IPUBES) *N* = 67	*p* value
Primiparity	27 (69.2%)	42 (62.7%)	0.496
Frank breech presentation	30 (76.9%)	48 (71.6%)	0.552
Maternal BMI (mean ± SD)	22.8 ± 4.3	23.5 ± 4.4	0.350
Fetal birth weight (grams; mean ± SD)	3389 ± 418	3276 ± 398	0.160
Epidural anesthesia	28 (71.8%)	39 (58.2%)	0.162
5′ APGAR (mean ± SD)	9.4 ± 1.3	9.6 ± 0.8	0.471
Umbilical cord arterial pH (mean ± SD)	7.24 ± 0.08	7.19 ± 0.10	0.014
Umbilical cord arterial pH < 7.0	0 (0.0%)	1 (1.4%)	0.471
Cesarean section	18 (46.2%)	5 (7.5%)	< 0.0001

**Table 3:** Diagnostic test accuracy compared to vaginal examination

Predictive value: cesarean section	Negative sonographic examination (-IPUBES)	Negative vaginal examination
Sensitivity	78.3%	69.6%
Specificity	74.7%	75.9%
Positive predictive value	46.2%	44.4%
Negative predictive value	92.5%	90.0%
AUC logistic regression	0.765	0.727

To test the reliability of the sonographic examination, we performed a matched-pair analysis comparing the sonographic and vaginal examination results. We detected a mean difference of results of 0.012 with a standard deviation of 0.027. The upper 95% confidence interval was 0.065, the lower 95% confidence interval was − 0.014.

Cesarean section indications were not significantly different between groups: In –IUBES, there was 1 case of mother’s wish, 4 cases of non-reassuring fetal heart rate, and 13 cases of arrest in labor. In + IPUBES, there were 2 cases of non-reassuring fetal heart rate and 3 cases of arrest in labor leading to cesarean section. The rate of CS indications in the group of cases with a cesarean section was not significantly different in relation to IPUBES (data not shown).

### Secondary outcome parameters

Parity, fetal posture, maternal BMI, epidural anesthesia, and fetal birth weight were not significantly different between + IPBUES and −IPBUES group (Table 2). The 5 min APGAR score was not significantly different with a mean value of 9.4 in −IPUBES and 9.6 in + IPBUES group (*p* = 0.471, Table 2). There was no significant difference in the rate of an arterial umbilical cord pH below 7.0 (−IPUBES: 0; 0%; + IPUBES: 1, 1.4%; *p* = 0.471).

In order to see, if the sonographic examination predicts birth outcome in vaginal birth, we compared the subgroups after exclusion of cesarean sections, resulting in a group with negative sonographic examination (−vIPUBES) and positive sonographic examination (+ vIPUBES). The rates of manual assistance were not significantly different between −vIPUBES (52.4%) and + vIPUBES group (35.5%, *p* = 0.172, Table 4). Also, the rates of either manual head assistance (Frank Nudge) or manual assisted arm delivery (Louwen Maneuver) were not significantly different (Table 4). Duration of the second stage was not significantly different between groups (−vIPUBES: 84 min; + vIPUBES: 79 min, *p* = 0.727, Table 4). Birth duration in total was significantly longer in −vIPUBES with 533 min in comparison to 440 min in + vIPUBES (*p* = 0.011, Table 4). In maternal outcome parameters (maternal blood loss and perineal injury rates), there were no significant differences (See Table 4).

**Table 4:** Vaginal birth outcome related to sonographic examination result

Characteristic	Negative IPUBES in vaginal deliveries (−vIPUBES) *N* = 21	Positive IPUBES in vaginal deliveries (+ vIPUBES) *N* = 62	*p* value
Manual assistance	11 (52.4%)	22 (35.5%)	0.172
Frank nudge (manual head delivery)	9 (42.9%)	21 (33.9%)	0.459
Louwen maneuver (manual arm delivery)	3 (14.3%)	6 (9.7%)	0.557
Duration 2nd stage (minutes, mean ± SD)	84 ± 70	79 ± 74	0.727
Birth duration (minutes, mean ± SD)	533 ± 262	440 ± 439	0.011
Maternal blood loss (ml, mean ± SD)	338 ± 144	340 ± 212	0.816
Perineal injury	10 (47.6%)	29 (46.8%)	0.947

## Discussion

In order to implement vaginal breech deliveries in more obstetrical centers, easily applicable methods and tools for birth observation and outcome prediction are necessary. Ultrasound examination to observe birth progression and to determine fetal head position and descent is an established practice in vertex deliveries [19–27]. In order to develop a new method for delivery observation in birth out of breech presentation, we introduced a new ultrasound-based examination. We monitored the essential leading fetal part representative for birth progression—the fetal pelvis—trans-abdominally and determined whether the fetal buttocks had entered the maternal pelvic inlet or not at beginning of the second stage of labor. This examination was reliable and significantly associated with birth outcome in our study.

A negative intrapartum ultrasound breech engagement sign (fetal pelvis above the maternal pelvic inlet, -IPUBES) was significantly associated with cesarean section in our cohort (see Table 2) and predicted cesarean section in stage II of labor with a sensitivity of 78.3%, a specificity of 74.7%, and a negative predictive value of 92.5% (see Table 3). Positive predictive value was low with 46.2%. In clinical daily practice, a negative IPUBES shows a high risk for cesarean section. This means that the birth process probably needs optimization. The mother giving birth should be motivated to move the pelvis and be as much in an upright position as possible. But if a positive IPBUES is found, vaginal birth is most likely to happen. Sensitivity of a negative vaginal examination result was not significantly different from the sensitivity of −IPBES in a lack-of-fit analysis of test value (*p* = 0.536). The sonographic examination proved to be reliable with a mean difference of only 0.012 in a matched-pair analysis comparing sonographic to vaginal examination results. Sonographic fetal descent assessment as shown in this manuscript can be used as reference for vaginal examination. In breech delivery, the most important indicator for birth progression is the fetal pelvis because it is the most massive leading structure in the birth canal. Birth progression observation thus should be focused on the fetal buttocks. This is in contrast to a study reporting on the prognostic value of the ‘breech progression angle’, where only the lowest reaching part of the fetus is detected [28].

The IPBUES was not associated with most birth outcome parameters but the cesarean section rate. Indicators for fetal morbidity such as the 5 min APGAR score or umbilical cord arterial pH < 7.0 were not significantly different (Table 2). Mean umbilical arterial pH was significantly lower in + IPUBES which is associated with a higher rate of vaginal birth in this group but not increased fetal morbidity (Table 2). There was no significant association between the examination result and maternal outcome, indicated by analysis of maternal blood loss and perineal injury as surrogate parameters (Table 4). The probability to need manual assistance in vaginal deliveries also was not significantly associated with IPBUES (Table 4). Vaginal birth out of breech presentation is a safe delivery mode, even if cesarean section or manual assistance is of need in the process [12, 13, 17]. Hence, affected fetal or maternal morbidity was not expected. Of note, overall birth duration but not duration of stage II was significantly longer in cases with a negative IPUBES (Table 4). This means that in delayed delivery, the fetal leading part seems to enter the maternal pelvis later and/or slower. This result may be biased by the proportion of primiparous women, which is slightly (but not significantly) higher in the group with a negative IPUBES (Table 2). In a systematic review analyzing intrapartum ultrasound in cephalic deliveries, the prognostic value to predict successful vaginal operative delivery is questioned [29]. The only study investigating fetal descent with ultrasound in breech presentation studies the inter-observer reliability of a sonographic evaluation of fetal descent [28]. Obviously, there is more research on breech delivery and intrapartum ultrasound necessary in order to document the value of such tests. Here we were able show that the examination of fetal pelvic descent is associated with the delivery mode. Through the intrapartum ultrasound examination presented in this study, the observer is able to detect birth arrest early and without error-prone-based utilities. Once detected, optimizing measures can be undertaken (e.g. upright maternal position, pelvic movement through walking). We hereby introduce a new and reliable method for delivery observation and birth outcome prediction which is quickly done and easily applicable. Additionally, this method enables physicians and obstetricians to train and verify their clinical vaginal examination.

### Strengths and limitations

The fixed time point of the examination is a disadvantage of this study because birth is a dynamic process. It was necessary to pinpoint the analyzed test in order to enable comparability and to exclude observational bias. In clinical daily practice, this ultrasound examination can be performed repeatedly in stage II, thus monitoring fetal descent/protrusion. Our newly introduced ultrasound examination might be used in a more flexible, time-dependent way in daily practice. We did not use a calculation or line-based system to analyze images. Other publications describe an infrapubic or suprapubic line for birth descent orientation [30, 31]. Sometimes, when the transducer is placed perpendicular strictly on the symphysis, fetal indicators for descent are not displayed and it is necessary to shift the transducer onto the pubic crest. Therefore, a line-based evaluation was difficult to implement. A line-based approach should be implemented in future in order to unify the examination. The outreach and impact of this study might be limited because it is a single-center study. A new examination method might lower barriers, hindering obstetrical centers from engaging in offering vaginal breech birth, but in order to spread competence in delivery attendance training, motivation, and gathered experience are obligatory.

## Conclusions

With this study, we introduce a new sonographic tool for breech birth observation. A high probability for emergency cesarean section can be diagnosed early with a negative intrapartum ultrasound breech engagement sign, verifying clinical vaginal examination results. A positive intrapartum ultrasound breech engagement sign is associated with vaginal birth. This new method of birth progression observation might be highly beneficial for spreading the management of vaginal intended birth out of breech presentation.

## Data Availability

Data is available upon request.
